# Soy germ extract alleviates menopausal hot flushes: placebo-controlled double-blind trial

**DOI:** 10.1038/s41430-018-0173-3

**Published:** 2018-05-30

**Authors:** Martin Imhof, Anca Gocan, Marianne Imhof, Mathias Schmidt

**Affiliations:** 1 Department of Gynecology and Obstetrics, Univ.-Doz. Dr. med. Martin Imhof, Landesklinikum Weinviertel, Wiener Ring 3-5, A-2100 Korneuburg, Austria; 2 Dr. med. Anca Gocan, Ärztezentrum Rahlgasse, Rahlgasse 1/12, A-1060 Wien, Austria; 3 Dr. Marianne Imhof, Life Research Technologies GmbH, Schönlanterngasse 11/13, A-1010 Wien, Austria; 4 Dr. Mathias Schmidt, Herbresearch Germany, Wartbergweg 15, 86874 Mattsies, Germany

**Keywords:** Endocrine system and metabolic diseases, Nutrition

## Abstract

**Background/objectives:**

A double-blind, placebo-controlled study was performed to assess the potency of a soy germ preparation for the alleviation of menopausal hot flushes.

**Subjects/methods:**

Caucasian women with at least seven hot flushes daily were treated with soy germ extract (100 mg isoflavone glycosides) daily or with placebo for 12 weeks, followed by 12 weeks of open treatment with soy. Outcome parameters were the number of hot flushes and the evaluation of the Greene Climacteric Scale.

**Results:**

A total of 192 women were included. As the hot flush diaries from one study centre were lost, the assessment of hot flushes was based on 136 participants (soy: 54 women; placebo: 82 women). After 12 weeks, 180 women were available for the analysis of Greene Scale and safety (soy and placebo: each 90 women). Hot flushes were reduced by 43.3% (–3.5 hot flushes) with soy and by 30.8% with placebo (–2.6; *p* < 0.001). After the open treatment phase with soy, both original groups showed a reduction of 68% of hot flushes. A subgroup analysis showed better effects for soy when symptoms were classified as “severe” at baseline. After 12 weeks of double-blind treatment, there was an improvement from baseline values of 71 and 78% with soy with the items “hot flushes” and “sweating”, compared with 24% for both items with placebo. Hormonal safety parameters remained uninfluenced.

**Conclusions:**

Soy germ extract with 100 mg of isoflavone glycosides was shown to modestly, but significantly reduce menopausal hot flushes.

## Introduction

The health-related effects of soy preparations are mostly attributed to the fraction of isoflavones, with genistein, daidzein and, as a relatively minor compound, glycitein as the most prominent representatives [1]. Isoflavones have been considered safe by the European Food Safety Authority with respect to effects at hormone-sensitive tissues such as breast or uterus with daily doses up to at least 150 mg [2, 3]. This conclusion on safety is backed by epidemiological observations of an association of reduced breast cancer risk with increasing dietary intake of isoflavones [4].

One of the earliest health-related observations associated with isoflavone exposure is the inverse relation between soy exposure in Asian societies and the incidence of menopausal hot flushes [5]. There is still some debate with respect to the effect size and its applicability to Western women with no life-long exposure to soy, and to the effect size in clinical trials. The North American Menopause Society (NAMS) concluded that soy-derived isoflavones are modestly effective in relieving menopausal symptoms [6]. The NAMS did, however, also point to a poor inter-study comparability, especially with respect to natural food and supplements containing isoflavones. Pharmacokinetic aspects may play a crucial role, as do the sample size in the studies and the number of daily hot flushes. The review by NAMS calculated a decrease of daily hot flushes frequency by 24–60% with isoflavone doses of 40–160 mg per day [6]. The US Food and Drug Organisation recommends a minimum of seven hot flushes daily for the performance of studies of menopausal vasomotor complaints [7]. It has been maintained that the beneficial effect of isoflavone supplementation on menopausal vasomotor complaints was consistently demonstrated in all clinical trials with an adequate design [8]. The effect could be verified in meta-analyses and reviews [9–17]. A relatively recent meta-analysis of 10 studies reporting hot flush frequencies indicated that isoflavone exposure results in a significantly greater reduction in hot flush frequency compared with placebo (pooled mean difference 0.89, *p* < 0.005) [9]. Soy isoflavones have been proposed as a first approach in the treatment of menopausal vasomotor symptoms [18], especially in women who cannot or do not want to be treated by hormone replacement therapy (HRT).

Genistein seems to play an important role [19]. Whereas the efficacy of genistein-rich preparations has been demonstrated, the applicability of soy germ preparations, which are richer in daidzein than in genistein, still had to be re-confirmed [2]. The aim of this study was therefore to examine the efficacy and safety of a daidzein-rich soy germ preparation against menopausal hot flushes. The primary endpoint was the number of hot flushes documented in a diary; the main secondary endpoints were differences in the Greene Climacteric Scale (GCS) and safety parameters. The present study was already performed in the years 2005–2006, but could not be published earlier for regulatory reasons under EU rules for proprietary data in the application for health claims.

## Materials and methods

The study was designed as a 12-week randomised, placebo-controlled double-blind parallel group trial followed by a 12-week open phase with all participants taking soy extract. It was performed in three study centres located in Vienna (Austria), in Alba Iulia (Rumania) and in Berlin (Germany) from January 2005 to November 2006.

### Vote of the ethics committee

The study was planned and carried out in accordance with the criteria of good clinical practice and the ethical standards defined in the declaration of Helsinki. An approval of the ethics committees of the three study centres was obtained, the regulatory authorities were properly informed.

### Study preparation

The study preparation was a commercially available food for special medical purposes (Alsitan GmbH, Greifenberg, Germany). Each capsule contained 250 mg of soy germ dry extract with 100 mg of total isoflavones (corresponding to 60 mg of isoflavone aglycones; with an average of 3.1% genistein, 15.5% daidzein and 7.7% glycitein per capsule). Further constituents were vitamins, minerals and trace elements.

Placebo consisted of externally undistinguishable capsules with microcrystalline cellulose.

Both preparations were administered at the dose of one capsule daily, to be administered in the morning with breakfast. Participant allocation to soy or to placebo was fully blinded.

### Inclusion and exclusion criteria

Inclusion of peri- and postmenopausal Caucasian women aged 45 to <70 years and suffering from natural hot flushes was made based on a telephone interview with a structured questionnaire, followed by a medical examination prior to inclusion. The main inclusion criterion was the presence of at least seven hot flushes daily, or at least 49 incidents per week, indicated by the patients themselves in the screening process.

HRT or the use of soy, red clover or black cohosh preparations within 6 months prior to study enrolment was not permitted, as was the use of hormonal contraceptives, oral medication causing or influencing hot flushes (e.g., clonidine, SSRI, SRNI, etc.) or the use of antibiotics within 3 months prior to enrolment. Additional vitamin or mineral supplement intake was excluded, as was the consumption of soy food with ≥1 portion per week. Vegetarians or participants with a soy allergy could not be included.

### Study parameters

After screening, women started a run-in period of 2 weeks with documentation of hot flushes in a diary. This documentation was continued throughout the study. The confirmatory part of the study referred to the demonstration of a statistical difference between groups for the reported number of hot flushes derived from the patient diaries after 12 weeks of double-blind treatment.

Additional, non-confirmatory comparisons were made for

- Subgroup analysis of hot flushes in the double-blind phase for women with <7 versus ≥7 hot flushes daily at baseline;
- Frequency of hot flushes in the 12-week open follow-up phase with soy;
- Differences in GCS scores between groups after 4, 8 and 12 weeks of double-blind treatment. The GCS adopted for this trial consisted of 21 items, rated on a scale of 0–3 with: 0 = not present, 1 = mild, 2 = moderate and 3 = severe (see Results for details). Subgroup evaluations were made for women with an individual GCS item score assessed as “severe” at baseline, and for those with ≥7 hot flushes daily, provided that the size of the resulting subgroup was at least 10 women in both treatment arms to assure meaningful results;
- Differences in GCS scores after 12 weeks of open follow-up treatment with soy;
- Safety parameters: group differences for clinical laboratory parameters, haematology and vaginal cytology, measured at baseline, at the end of the double-blind phase and again at the end of the open treatment phase.

Adverse events and compliance were actively monitored through telephone contacts in weeks 2, 6, 10, 16 and 20, and during the visits in weeks 4, 8, 12 and 24. They were assessed for causality with the study preparation, and compared between groups.

For missing values, the “last observation carried forward” (LOCF) method was planned.

### Case number calculation

The assumptions for the case number calculation were a placebo effect of 25% reduction of hot flushes, a verum effect at least 15% above placebo, a power of 90% and an alpha error of 5%. Including an estimated drop-out rate of 20%, the calculated minimum number of study participants was 120.

### Statistics

The statistical analysis was based on a between-group comparison of the frequency of hot flushes, with the null hypothesis expecting no difference between soy and placebo. Patients were assigned a consecutive random number on a random list in blocks of 10 according to their entry into the trial. Unblinding took place after the documentation was completed and the database was closed. A stratification according to menopausal status (peri-menopausal versus postmenopausal) was a pre-defined option.

Significances in intergroup differences for hot flush frequency and GCS parameters were calculated using the Mann–Whitney *U*-test and the Wilcoxon W test. Normal distribution of demographic data at baseline and potential group differences were statistically examined using the Kolmogorov–Smirnov test. The threshold for significance was defined on the 5% level for the evaluation of the hot flushes. As the Mann–Whitney *U*-test used for the group comparisons of the GCS parameters is highly sensitive even to minor changes, the threshold for significance was set to 1% for this parameter. Comparisons of frequencies of adverse effects between groups were made with the Fisher exact test, with a defined threshold for statistical significance of 3%. All tests were performed using SPSS v12.

## Results

### Study population in the 12-week double-blind treatment (phase I)

**Fig. 1:**
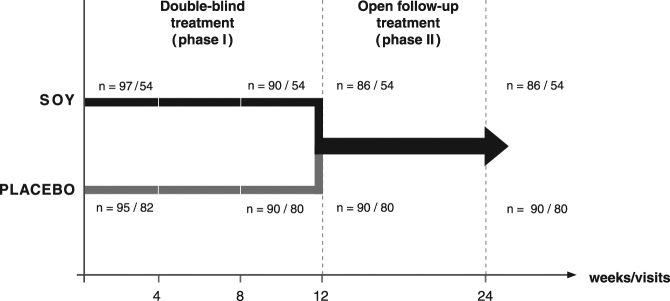
Flow chart for the double-blind and the open treatment phases. Group population is indicated for the safety population and the sub-population with hot flush diaries

A total of 192 healthy women with menopausal symptoms was included into the trial (soy: *n* = 97; placebo: *n* = 95; Fig. 1). As all women were examined for safety of application, this population was considered the safety population.

Due to a major communication error, the women examined in Rumania had not filled in a patient diary for hot flushes. Data sets from women having filled in hot flush diaries were therefore not available for all 192, but only for a subgroup of 136 women with, in addition, an uneven repartition of patients per group (soy: *n* = 54, placebo: *n* = 82 at study start; Fig. 1). This smaller number of women was the population for the analysis of data on the confirmatory parameter, the frequency of hot flushes.

### Drop-outs in phase I

Twelve participants prematurely terminated study participation during the double-blind phase (soy: *n* = 7; placebo: *n* = 5). In four of the five cases of drop-outs in the placebo group, the indicated reason was “lack of efficacy”, and in one case no specific reason was given. One of the women indicating no effect with placebo had also reported diarrhoea as an adverse event. The latter women and another participant who had indicated “lack of efficacy” upon termination after the visit at week 8 had filled in a patient diary on hot flushes, thus reducing the population for the analysis of the frequency of hot flushes from 82 to 80.

In the soy group, one woman stopped treatment for lack of efficacy, one had to terminate due to travelling, and five did not indicate a specific reason. None of the women terminating treatment belonged to the subgroup of women with data from patient diaries on hot flushes, thus the number of participants with data for the analysis of the primary parameter remained unchanged at 54.

Correspondingly, after 12 weeks there were 180 sets of Phase I data available (per protocol safety population as displayed in Fig. 1 for the time point 12 weeks; soy and placebo both *n* = 90), including a total of 134 patients with hot flush diaries for the confirmatory testing (soy; *n* = 54, placebo: *n* = 80).

### Study population in the 12-week open follow-up treatment with soy extract (phase II)

Four of the 180 participants regularly terminating study phase I (Fig. 1) opted not to continue into the open trial phase. All four women were originally from the soy group, and none had filled in a patient diary for hot flushes. The population for the open follow-up phase II with a 12-week treatment with soy extract was therefore *n* = 176 for the Greene Scale and the safety parameters (Fig. 1: former soy group *n* = 86; former placebo group *n* = 90), and *n* = 134 for the analysis of hot flushes in the patient diaries (Fig. 1: soy: *n* = 54, placebo: *n* = 80). There were no further drop-outs during the open treatment phase.

### Demographic data

Comparability of groups was given at baseline. The only statistically significant differences between groups were found for estradiol and testosterone levels, the number of reticulocytes, transferrin, triglycerides and gamma-glutamyl transpeptidase. In all cases, the difference was only minor, clinically not important and well within the physiological range (tabulated data shown in supplementary online material for women completing the 24 weeks of treatment).

### Confirmatory primary efficacy parameter: hot flushes

Patient diaries were evaluated at the end of the study. Although all participants had indicated during screening to suffer from at least seven hot flushes per day, the patient diaries showed that in 23 out of 136 women assessed for hot flush frequency (two later drop-outs included) the number of hot flushes was in fact lower at baseline. There was, however, no significant difference between groups for hot flush frequency at baseline in the full group and the subgroups with <7 or ≥7 hot flushes daily (see tabulated baseline data in supplementary online data).

### Reduction of hot flushes

**Fig. 2:**
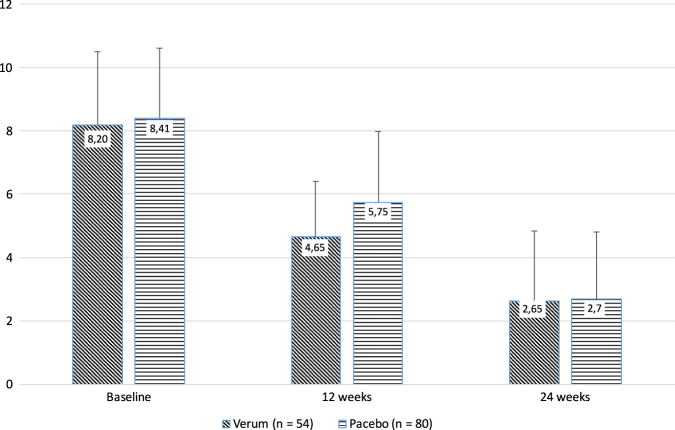
Reduction of hot flush frequency during the 12-week placebo-controlled phase followed by 12 weeks of open treatment with exposure to soy extract

At the end of the double-blind study phase, the number of hot flushes in the population with patient diaries (Fig. 1) was reduced from 8.2 ± 2.3 to 4.7 ± 1.8 (–43.3%) in the soy group (*n* = 54), and from 8.4 ± 2.2 to 5.8 ± 2.3 (–30.8%) in the placebo group (*n* = 82; Mann–Whitney *U*-test, *p* < 0.001; Fig. 2).

Results were practically identical when calculated for the per protocol population, that is, without the data of women with an early study termination (drop-outs) and therefore without using the LOCF method: *n* = 54 women in the soy group (results unchanged), and *n* = 80 women in the placebo group, the latter population showing a score reduction from 8.4 ± 2.2 to 5.8 ± 2.2 (–31.7%). There were no missing values.

The improvement of hot flush frequency continued in both groups into the open trial phase II, when all women were exposed to soy extract. In the former soy group (*n* = 54, Fig. 1), the reduction reached 67.7% of baseline (2.7 ± 2.2; Fig. 2). The former placebo group now openly treated with soy (*n* = 80, Fig. 1) caught up, reaching the same level as the former soy group with 67.9% of baseline (2.7 ± 2.1; no statistical difference between the two groups: *p* = 0.768; Fig. 2).

### Subgroup analysis: population with ≥ 7 hot flushes daily

**Fig. 3:**
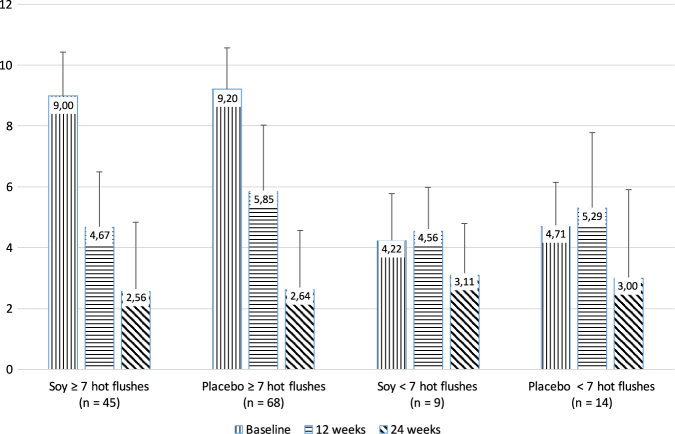
Subgroup analysis for women with ≥7 versus ≤6 daily hot flushes at baseline: Reduction of hot flush frequency during the 12-week placebo-controlled phase followed by 12 weeks of open treatment with soy extract

Whereas the superiority of verum over placebo was confirmed for the women with seven and more hot flushes, the effect was lost in the women with fewer vasomotor incidents with no difference between soy and placebo (Fig. 3, Table 1).

**Table 1:** Reduction of hot flushes, calculated for the per protocol population (excluding drop-outs)

		All women		Subgroup ≥ 7 hot flushes		Subgroup ≤ 6 hot flushes	
		Soy	Placebo	Soy	Placebo	Soy	Placebo
Baseline	*n*	54	80	45	66	9	14
	Number	8.20 ± 2.30	8.44 ± 2.19	9.00 ± 1.43	9.20 ± 1.37	4.22 ± 1.56	4.71 ± 1.44
	*p*	0.619 (n.s.)		0.458 (n.s.)		0.477 (n.s.)	
12 Weeks	*n*	54	80	45	66	9	14
	Number	4.65 ± 1.75	5.75 ± 2.23	4.67 ± 1.82	5.85 ± 2.18	4.56 ± 1.42	5.29 ± 2.49
	Reduction	43.3%	31.6%	48.1%	36.4%	-8.1%	-12.3%
	*p*	0.001		0.001		0.439 (n.s.)	
24 Weeks	*n*	54	80	45	66	9	14
	Number	2.65 ± 2.19	2.70 ± 2.11	2.56 ± 2.28	2.64 ± 1.93	3.11 ± 1.69	3.00 ± 2.91
	Reduction	67.5%	67.9%	71.6%	71.3%	26.3%	36.3%
	*p*	0.786		0.485		0.516 (n.s)	

Negative percentage improvements reflect an increased number of hot flushes versus baseline. The *p*-values are for group differences at baseline, after 12 and 24 weeks (Mann–Whitney test)

### Secondary efficacy parameter: GCS

There was no statistically significant group difference for any of the items, scores and subscores of the GCS at the start of the study (Table 2). With the exception of the early study terminations, there were no missing items in the data. Statistical analyses were made for both, the full intention-to-treat population and the per protocol completers. The differences for the two statistical evaluations were only minor. The data in Table 2 are therefore presented for the per protocol population (excluding early study terminations).

**Table 2:** Improvements of the symptoms of the Greene Climacteric Scale in score values (per protocol population with *n* = 86 patients in the soy group and *n* = 90 patients in the placebo group)

	Baseline		Week 12		Week 24	
Item	Soy	Placebo	Soy	Placebo	Soy	Placebo
1. Tachycardia/palpitations	1.31 ± 0.88	1.33 ± 0.97	0.55 ± 0.64*	0.97 ± 0.93	0.31 ± 0.62	0.28 ± 0.58
2. Feeling tense or nervousness	1.97 ± 0.83	1.88 ± 0.93	0.56 ± 0.75*	1.60 ± 1.09	0.56 ± 0.83	0.37 ± 0.59
3. Difficulty in sleeping	2.22 ± 0.95	2.13 ± 0.93	0.60 ± 0.94*	1.90 ± 1.10	0.50 ± 0.90	0.43 ± 0.85
4. Excitable	1.98 ± 0.91	2.01 ± 0.93	0.62 ± 0.86*	1.74 ± 1.14	0.53 ± 0.89	0.37 ± 0.57
5. Panic attacks	1.45 ± 1.13	1.27 ± 1.27	0.30 ± 0.69*	1.21 ± 1.24	0.27 ± 0.62	0.11 ± 0.35
6. Difficulty in concentrating	1.93 ± 0.98	1.67 ± 1.04	0.53 ± 0.81*	1.63 ± 1.16	0.47 ± 0.71	0.37 ± 0.61
7. Feeling tired or lacking energy	1.92 ± 0.91	1.67 ± 0.95	0.58 ± 0.83*	1.77 ± 1.13	0.41 ± 0.64	0.32 ± 0.58
8. General loss of interest in most things	1.26 ± 1.04	1.28 ± 1.02	0.42 ± 0.64*	1.33 ± 1.16	0.38 ± 0.71	0.22 ± 0.47
9. Feeling unhappy or depressed	1.57 ± 1.11	1.62 ± 1.11	0.37 ± 0.67*	1.49 ± 1.27	0.36 ± 0.65	0.20 ± 0.48
10. Crying spells	1.01 ± 0.98	0.99 ± 0.95	0.22 ± 0.52*	1.00 ± 1.06	0.24 ± 0.68	0.30 ± 0.55
11. Feeling perturbed (inner tension)	1.92 ± 1.01	1.80 ± 0.96	0.53 ± 0.79*	1.74 ± 1.14	0.45 ± 0.73	0.37 ± 0.57
12. Feeling dizzy or faint	1.07 ± 0.86	0.87 ± 0.94	0.51 ± 0.72*	0.89 ± 0.92	0.31 ± 0.56	0.26 ± 0.55
13. Pressure or tightness in head or body	1.09 ± 1.04	0.87 ± 0.97	0.43 ± 0.66*	0.84 ± 0.94	0.26 ± 0.46	0.14 ± 0.38
14. Parts of body feeling numb or tingling	0.72 ± 0.95	0.60 ± 0.83	0.36 ± 0.72	0.54 ± 0.86	0.31 ± 0.67	0.16 ± 0.36
15. Headaches	1.01 ± 0.86	1.04 ± 1.02	0.48 ± 0.76*	0.90 ± 0.92	0.30 ± 0.65	0.29 ± 0.52
16. Muscle or joint pains	1.60 ± 0.96	1.56 ± 1.07	0.59 ± 0.83*	1.21 ± 1.14	0.38 ± 0.75	0.43 ±± 0.86
17. Loss of feeling in hands or legs	0.58 ± 0.90	0.74 ± 0.91	0.33 ± 0.71	0.54 ± 0.77	0.34 ± 0.76	0.09 ± 0.29
18. Breathing difficulties	0.60 ± 0.92	0.53 ± 0.84	0.26 ± 0.62	0.43 ± 0.70	0.16 ± 0.48	0.09 ± 0.29
19. Hot flushes	2.59 ± 0.66	2.58 ± 0.65	0.72 ± 0.88*	2.02 ± 1.07	0.63 ± 0.92	0.66 ± 0.84
20. Sweating at night	2.43 ± 0.78	2.32 ± 0.85	0.66 ± 0.85*	1.90 ± 1.08	0.55 ± 0.86	0.56 ± 0.82
21. Loss of interest in sex	2.08 ± 1.04	1.88 ± 1.08	0.67 ± 0.94*	1.80 ± 1.19	0.63 ± 1.02	0.41 ± 0.78

Group comparisons: *p ≤ 0.005

The first interim visit after 4 weeks already showed significant group differences in favour of soy for the parameters excitability and hot flushes. After 8 weeks of double-blind treatment, there were significant group differences for all psychological items of the GCS (items 1–11) with the exception of item 1 (tachycardia/palpitations). Of the somatic symptoms (items 12–18), only item 16 (muscle or joint pain) reached statistical significance. Vasomotor and sexual symptoms (items 19–21) showed a statistically significant group difference in favour of soy (Mann–Whitney *U*-test, *p* < 0.01, data not shown).

After 12 weeks of double-blind treatment all parameters, with the exception of three somatic symptoms (body parts feeling numb or tingling, loss of feeling in hands or legs, breathing difficulties), were significantly different between groups (*p* < 0.01; Table 2). With the open treatment phase under soy, the differences vanished through an improvement in the former placebo group to the level of the former verum group.

The effects became even more evident when the single items of the Green Climacteric Scale were assessed for women with individual symptoms rated as “severe” at baseline. Table 3 shows the reduction versus baseline after 4 and 12 weeks of double-blind treatment for all patients, and for the subgroup of patients where the item was assessed as “severe” at baseline.

**Table 3:** Improvements of the symptoms of the Greene Climacteric Scale in percent of baseline values

Symptom		Soy/placebo, *n*	Soy		Placebo	
Week			4	12	4	12
1. Tachycardia/palpitations	TG SG	86/90 7/12	14% n.e.	58% n.e.	13% n.e.	28% n.e.
2. Feeling tense or nervousness	TG SG	86/90 26/26	16% 41%	72% 75%	9% 21%	15% 31%
3. Difficulty in sleeping	TG SG	86/90 44/39	21% 31%	73% 75%	11% 17%	11% 26%
4. Excitable	TG SG	86/90 39/32	19% 36%	69% 74%	0% 10%	13% 27%
5. Panic attacks	TG SG	86/90 20/24	30% 38%	79% 77%	18% 22%	4% 31%
6. Difficulty in concentrating	TG SG 1	86/90 30/25	29% 51%	72% 81%	1% 16%	2% 24%
7. Feeling tired or lacking energy	TG SG	86/90 26/20	29% 46%	70% 76%	5% 10%	6% 25%
8. General loss of interest in most things	TG SG	86/90 12/12	28% 42%	67% 81%	6% 25%	4% 36%
9. Feeling unhappy or depressed	TG SG	86/90 23/25	35% 58%	76% 83%	14% 24%	8% 32%
10. Crying spells	TG SG	86/90 6/6	15% n.e.	78% n.e.	2% n.e.	1% n.e.
11. Feeling perturbed (inner tension)	TG SG	86/90 32/26	25% 48%	72% 79%	6% 13%	3% 17%
12. Feeling dizzy or faint	TG SG	86/90 5/6	27% n.e.	52% n.e.	12% n.e.	3% n.e.
13. Pressure or tightness in head or body	TG SG	86/90 10/8	30% n.e.	61% n.e.	13% n.e.	3% n.e.
14. Parts of body feeling numb or tingling	TG SG	86/90 7/3	10% n.e.	50% n.e.	4% n.e.	9% n.e.
15. Headaches	TG SG	86/90 5/9	1% n.e.	53% n.e.	5% n.e.	14% n.e.
16. Muscle or joint pains	TG SG	86/90 14/20	28% 19%	63% 67%	11% 18%	22% 37%
17. Loss of feeling in hands or legs	TG SG	86/90 4/4	2% n.e.	44% n.e.	21% n.e.	27% n.e.
18. Breathing difficulties	TG SG	86/90 5/2	13% n.e.	58% n.e.	0% n.e.	19% n.e.
19. Hot flushes	TG SG	86/90 59/59	23% 27%	72% 71%	5% 7%	22% 24%
20. Sweating at night	TG SG	86/90 50/48	24% 28%	72% 78%	9% 14%	18% 24%
21. Loss of interest in sex	TG SG	86/90 40/34	22% 31%	68% 74%	9% 31%	4% 10%

*TG* total group, *SG* subgroup with GCS value = 3 for the corresponding item, *n.e*. not evaluated due to patient numbers < 10 in the soy or the placebo arm

Specifically, in the high severity subgroup there was an improvement for the parameters “hot flushes” (item 19) and “nightly sweating” (item 20) by 71% and 78% with soy, and by 24% and 24% with placebo. “Loss of interest in sex” (item 21) was improved by 74% with soy and by 10% with placebo. Furthermore, there were distinct differences between soy and placebo for the psychological symptoms “feeling tense or nervous” (item 2), “difficulty in sleeping” (item 3), “difficulty in concentrating” (item 6), “feeling tired or lacking energy” (item 7), “general loss of interest” (item 8), “feeling unhappy or depressed” (item 9) and “inner tension” (item 11), with in all cases reductions from baseline values of at least 75% with soy. Soy had generally less effects on the somatic symptoms.

### Vital signs and clinical laboratory findings including safety parameters

All clinical and safety laboratory parameters measured at baseline were also taken after 12 and 24 weeks. Results for parameters where at one or more points in the study a significant group difference was encountered are shown online in a supplementary table. None of the changes was rated as clinically important, as all were within the physiological range.

In blood analysis, a significant increase of iron levels was found after 24 weeks of exposure (*p* = 0.007), paralleled by a tendency towards improved values of ferritin, haematocrit, haemoglobin, erythrocyte and reticulocyte count after 24 weeks.

### Safety and adverse events

Mild adverse events were reported by 13 study participants during the study (Table 4). Three adverse events with an at least possible relationship with soy extract were reported: one case of diarrhoea, one case of transient skin blemishes and one case of heartburn.

**Table 4:** Adverse events

Event	Causality assessment
Verum	
2 × Diarrhoea in the double-blind phase, but no longer with open treatment	Unrelated
Diarrhoea in the double-blind and the open phase	Possible
Skin blemishes in week 2–3, then spontaneous recovery	Possible
Heartburn in the open phase	Possible
Placebo	
4 × Diarrhoea in the double-blind phase, but no longer with open treatment (1 × drop-out)	Unrelated
Diarrhoea in the double-blind phase with placebo and during open treatment with verum	Unrelated
2 × Heartburn in the double-blind phase with placebo and in the open phase with verum	Unrelated
Flu-like infection in the double-blind phase with placebo, relapse in week 20 during open treatment with verum	Unrelated

There was a tendency towards a reduction of liver function parameters found slightly elevated at baseline (data not presented). Soy had no adverse effect on hormonal parameters such as the proliferation of the vaginal endothelium or the sexual hormones estradiol, prolactin, SHBG and FSH. Similarly, there was no effect on thyroidal parameters.

## Discussion

### Effects on menopausal symptoms

The study resulted in the observation of a reduction of hot flushes by 43.3% with soy germ extract and 30.8% with placebo under 12 weeks of double-blinded exposure, and by 68% after open continuation for another 12 weeks. Further confirmation of benefits of soy germ extract came from the descriptive analysis of effects documented through the GCS.

The placebo effect of 30.8% reduction of hot flushes was in the range of what was expected from published studies, but the soy effect of 43.3% reduction was lower than anticipated from the general experience with the preparation.

Several clinical double-blind trials reported reductions of hot flushes with similar exposure to soy isoflavones or isolated genistein and a comparable severity of symptoms at baseline [20–23]. Improvements from baseline with the isoflavone-containing preparation ranged from 44 to 68%, whereas improvement with placebo was found in the range of 10 to 42%. Due to differences in design and duration the studies can hardly be compared. They do, however, still add plausibility to the findings in this trial with respect to effect size. Overall, isoflavones are clearly less performant than HRT, which typically reduces the number of menopausal hot flushes by 70–77% [24–26].

Whereas the difference in hot flushes between soy and placebo at the end of the double-blind phase may not seem highly impressive (approximately one event daily in favour of soy), the women in real life will also profit from the placebo effect. The observed reduction of the number of hot flushes is expected to be a tangible relief of burden for the women having these symptoms, and therefore to be of clinical importance.

### Discussion of safety

The established long-term safety of soy preparations makes isoflavones a suitable first-line approach especially for women who do not want to or cannot use HRT [18].The safety parameters controlled in this trial confirm the lack of a potentially undesired effect on hormone-sensitive tissues. The observation of a significant improvement of iron levels and a tendency of improved values of ferritin, haematocrit, haemoglobin, erythrocyte and reticulocyte count after 24 weeks of exposure is not surprising, as the study preparation also supplemented this element (5 mg/day). The exposure to the soy preparation had no effect on blood pressure and heart rate, as confirmed in a recent clinical double-blind trial with postmenopausal women exposed to soy and daidzein [27].

The observations with respect to adverse events cannot be attributed to soy germ extract as such, as the study preparation also contained vitamins and trace elements, some of which (such as iron) may cause gastrointestinal complaints. The occurrence of diarrhoea and heartburn was also observed during placebo treatment and might rather be related to the general health situation of the patients than to treatment with the study preparation.

### Factors potentially impacting clinical outcomes

Several issues with a potentially negative impact on the study outcome or interpretation were identified during the evaluation of the study: the lost patient diaries, the number of hot flushes at inclusion and the application of the “LOCF” method. The communication error leading to the non-availability of patient diaries from 29.2% of the study participants and the retrospective observation that 17.2% of the women who provided data on hot flushes had less than seven hot flushes daily at baseline may have caused a negative impact on statistical power and on the potential effect size.

The impact of the number of hot flushes at baseline has not been confirmed in a meta-analysis [19], although the authors state that this contradiction to published trials may have been caused by their rather high cut-off value of baseline hot flushes. Other studies (e.g., Messina and Hughes [14]) found a correlation of effect strength with the number of hot flushes at baseline, and the US-American Food and Drug Administration recommended a minimum of seven hot flushes daily as a criterion for patient inclusion [7]. The issue is still under debate, but the subgroup analysis performed in our study would rather confirm the existence of such a better effect of soy preparations on menopausal hot flushes with higher numbers of daily hot flushes.

Finally, the method of LOCF may according to studies published after the performance of this trial today be critically viewed as a potential distortion of the results through an overestimation of the effect, especially in diseases where there is no spontaneous improvement of symptoms or potentially even a deterioration over time [28]. This might be a situation encountered with menopausal hot flushes, thus a modern study design would rather replace the LOCF procedure by multiple imputation methods. In this study, the comparison of outcomes with and without the LOCF method showed only minor or no differences for hot flush frequency, GCS and safety parameters. The use of the LOCF method should therefore not have created a noteworthy bias.

## Conclusions

Two conclusions can be drawn from the results: (a) even though there was an early effect on hot flushes with soy, the full effect was obviously not yet reached after 12 weeks, as the number of hot flushes still improved during the 12 weeks of open follow-up with soy; and (b) the effect size depends on the severity of symptoms, with better results achieved for higher numbers of daily hot flushes and for a higher severity of symptoms.

Overall, the study confirms a modest, but still statistically significant and clinically important efficacy of soy germ extract against menopausal vasomotor symptoms, despite the fact that the study was underpowered due to lower than anticipated group size for hot flushes. All studies published to date show that isoflavones are not a miracle cure for hot flushes, with approximately cutting the number of daily hot flushes by half.

## Electronic supplementary material

Supplementary Table 1

Supplementary Table 2
