# Supplementary material for: Soy germ extract alleviates menopausal hot flushes: placebo-controlled double-blind trial
Source: Eur J Clin Nutr. 2018 May 30;72(7):961–70. doi: 10.1038/s41430-018-0173-3 (PMC6035149; doi:10.1038/s41430-018-0173-3)
Supplement: Supplementary file 1 — Supplementary Table 1 [file 41430_2018_173_MOESM1_ESM.docx]

Supplementary Table 1: Demographic baseline data of women completing the 24 weeks of treatment (n = 176 for all parameters except hot flushes: n = 136). Missing values were not replaced, therefore the group size for the individual parameters may be smaller. Statistical differences were calculated using the One-sample Kolmogorov-Smirnov test on normal distribution, followed by the Mann-Whitney test. Differences for marital status were calculated with verbal rating scales.

| Parameter | All | Soy | | Placebo | p |
| --- | --- | --- | --- | --- | --- |
| Demographic parameters | | | | | |
| Age (years) | n = 146  53.9 ± 5.8 | | n = 61  54.3 ± 6.4 | n = 85  53.6 ± 5.3 | 0.590 (n.s.) |
| Size (cm) | n = 156  165.5 + 6.4 | | n = 78  166.2 ± 6.2 | n = 78  164.7 ± 6.6 | 0.169 (n.s.) |
| Body weight (kg) | n = 149  67.4 ± 11.9 | | n = 76  66.8 ± 11.6 | n = 73  68.0 ± 12.3 | 0.391 (n.s.) |
| Marital status:  Married  Divorced  Single | n = 154  65.6 %  21.5 %  13.0 % | | n = 77  62.3 %  24.7 %  13.0 % | n = 77  68.8 %  18.2 %  13.0 % | 0.471 (n.s.) |
| Number of children | n = 154  1.3 ± 1.1 | | n = 77  1.2 ± 1.1 | n = 77  1.4 ± 1.2 | 0.541 (n.s.) |
| Clinical parameters | | | | | |
| Blood pressure systolic (mm Hg) | n = 149  138.7 ± 15.4 | n = 76  138.6 ± 16.6 | | n = 73  138.8 ± 14.1 | 0.950 (n.s.) |
| Blood pressure diastolic (mm Hg) | n = 149  84.0 ± 7.9 | n = 76  83.8 ± 8.5 | | n = 73  84.2 ± 7.4 | 0.934 (n.s.) |
| Heart rate (beats per minute) | n = 149  72.3 ± 8.5 | n = 76  72.3 ± 8.3 | | n = 73  72.4 ± 8.7 | 0.459 (n.s.) |
| Hot flushes (per day) | n = 136  8.35 ± 2.23 | n = 54  8.20 ± 2.30 | | n = 82  8.44 ± 2.19 | 0.586 (n.s.) |
| 7 and more hot flushes daily | n = 111  9.12 ± 1.39 | n = 45  9.00 ± 1.43 | | n = 66  9.20 ± 1.37 | 0.458 (n.s.) |
| Less than 7 hot flushes daily | n = 25  4.52 ± 1.47 | n = 9  4.22 ± 1.56 | | n = 16  4.71 ± 1.44 | 0.477 (n.s.) |
| Hormonal parameters | | | | | |
| Estradiol (pmol/l) | n = 174  51.03 ± 83.8 | n = 86  40.1 ± 74.1 | | n = 88  61.7 ± 91.4 | 0.021 |
| Testosteron (nmol/l)  Norm: < 3.5 | n = 173  1.0 ± 0.5 | n = 86  0.9 ± 0.5 | | n = 87  1.0 ± 0.5 | 0.047 |
| Vaginal cytology (proliferation grade I to IV) | n = 116  2.4 ± 0.6 | n = 48  2.4 ± 0.6 | | n = 68  2.5 ± 0.6 | 0.852 (n.s.) |
| Prolactin (µg/l)  Norm: 3-19 | n = 174  9.3 ± 5.6 | n = 85  9.4 ± 5.5 | | n = 89  9.3 ± 5.6 | 0.718 (n.s.) |
| SHBG (nmol/l)  Norm: 30-95 | n = 174  51.9 ± 29.8 | n = 86  43.5 ± 16.8 | | n = 88  60.2 ± 36.8 | 0.009 |
| FSH (IU/L)  Norm: 2-100 | n = 175  78.1 ± 39.0 | n = 86  81.6 ± 35.7 | | n = 89  74.7 ± 41.9 | 0.279 |
| Blood cell counts | | | | | |
| Leucocytes (cells/nl)  Norm: 4-11 | n = 175  6.0 ± 1.5 | n = 85  6.9 ± 1.1 | | n = 90  6.9 ± 1.8 | 0.430 (n.s.) |
| Erythrocytes (cells/pl)  Norm: 3.9-5.4 | n = 175  4.4 ± 0.3 | n = 85  4.4 ± 0.3 | | n = 90  4.4 ± 0.3 | 0.858 (n.s.) |
| Hemoglobin (g/dl)  Norm: 12-16 | n = 175  13.6 ± 0.9 | n = 85  13.6 ± 0.9 | | n = 90  13.5 ± 0.9 | 0.511 (n.s.) |
| Hematocrit (%)  Norm: 35-47 | n = 175  40.1 ± 2.7 | n = 85  40.9 ± 2.5 | | n = 90  40.6 ± 2.9 | 0.596 (n.s.) |
| Thrombocytes (cells/nl)  Norm: 150-400 | n = 175  288.3 ± 56.1 | n = 85  289.1 ± 51.0 | | n = 90  287.4 ± 60.8 | 0.564 (n.s.) |
| Reticulocytes (%)  Norm: 7-15 | n = 174  10.1 ± 2.5 | n = 85  10.5 ± 2.5 | | n = 89  9.7 ± 2.5 | 0.031 |
| Lymphocytes (%)  Norm: 25-45 | n = 148  35.2 ± 7.9 | n = 75  35.1 ± 7.6 | | n = 73  35.3 ± 8.1 | 0.002 (n.s.) |
| Monocytes (%)  Norm: 3-7 | n = 148  6.0 ± 1.9 | n = 75  6.1 ± 1.6 | | n = 73  6.0 ± 2.3 | 0.763 (n.s.) |
| Eosinophilic leucocytes (%)  Norm: 1-4 | n = 148  2.0 ± 1.8 | n = 75  2.0 ± 1.5 | | n = 73  2.1 ± 2.0 | 0.686 (n.s.) |
| Basophilic leucocytes (%)  Norm: 0-1 | n = 148  1.0 ± 0.6 | n = 75  1.1 ± 0.6 | | n = 73  1.0 ± 0.6 | 0.531 (n.s.) |
| Electrolytes | | | | | |
| Sodium (mmol/l)  Norm: 135-145 | n = 175  140.4 ± 2.0 | n = 85  140.4 ± 1.9 | | n = 90  140.3 ± 2.1 | 0.578 (n.s.) |
| Potassium (mmol/l)  Norm: 3.4-5.2 | n = 175  4.0 ± 0.8 | n = 85  4.8 ± 0.8 | | n = 90  5.0 ± 0.8 | 0.132 (n.s.) |
| Iron (µg/l)  Norm: 7-28 | n = 176  9.8 ± 5.2 | n = 86  9.7 ± 5.3 | | n = 90  9.8 ± 5.2 | 0.861 (n.s.) |
| Transferrin (mg/dl)  Norm: 200-360 | n = 173  260.2 ± 38.5 | n = 85  251.0 ± 31.6 | | n = 88  269.1 ±42.5 | 0.002 |
| Ferritin (µg/ml)  Norm: 10-200 | n = 174  68.9 ± 52.5 | n = 86  69.3 ± 44.3 | | n = 88  68.5 ± 59.7 | 0.229 (n.s.) |
| Kidney, liver and thyroidal parameters | | | | | |
| Creatinin (mg/dl)  Norm: ≤ 1.1 | n = 176  0.81 ± 0.18 | n = 86  0.82 ± 0.19 | | n = 90  0.80 ± 0.18 | 0.458 (n.s.) |
| Blood urea nitrogen (mg/dl)  Norm: 14-46 | n = 176  17.5 ± 10.0 | n = 86  16.7 ± 8.1 | | n = 90  18.2 ± 11.6 | 0.537 (n.s.) |
| Total cholesterol (mg/dl)  Norm: < 250 | n = 176  231 ± 46 | n = 86  235 ± 50 | | n = 90  227 ± 42 | 0.266 (n.s.) |
| HDL (mg/dl)  Norm: > 45 | n = 176  70 ± 19 | n = 86  68 ± 18 | | n = 90  72 ± 20 | 0.214 (n.s.) |
| LDL (mg/dl)  Norm: ≤ 160 | n = 175  118 ± 34 | n = 86  116 ± 35 | | n = 89  120 ± 34 | 0.360 (n.s.) |
| Triglycerides (mg/dl)  Norm: < 180 | n = 175  156 ± 85 | n = 86  164 ± 75 | | n = 89  148 ± 93 | 0.046 |
| Bilirubin (mg/dl)  Norm: < 1.1 | n = 176  0.5 ± 0.3 | n = 86  0.6 ± 0.4 | | n = 90  0.5 ± 0.3 | 0.951 (n.s.) |
| AST (U/l)  Norm: < 35 | n = 172  26.4 ± 6.5 | n = 83  26.0 ± 5.6 | | n = 89  26.8 ± 7.2 | 0.793 (n.s.) |
| ALT (U/l)  Norm: < 36 | n = 170  24.8 ± 10.9 | n = 82  25.5 ± 11.0 | | n = 88  24.0 ± 10.7 | 0.198 (n.s.) |
| GGT (U/l)  Norm: < 40 | n = 158  23.5 ± 16.5 | n = 77  24.3 ± 12.0 | | n = 81  22.8 ± 19.9 | 0.043 |
| CRP (mg/dl)  Norm: < 8 | n = 176  3.6 ± 6.2 | n = 86  3.9 ± 5.9 | | n = 90  3.4 ± 6.4 | 0.838 (n.s.) |
| Glucose (mg/dl)  Norm: < 130 | n = 174  96.2 ± 23.2 | n = 85  97.9 ± 27.0 | | n = 89  94.6 ± 18.9 | 0.483 (n.s.) |
| TSH (mU/l)  Norm: 0.27-4.2 | n = 176  1.8 ± 1.1 | n = 86  1.7 ± 1.0 | | n = 90  1.8 ± 1.1 | 0.954 (n.s.) |
| T3 (µg/l)  Norm: 0.5-2.1 | n = 147  1.4 ± 0.3 | n = 74  1.5 ± 0.3 | | n = 73  1.4 ± 0.2 | 0.142 (n.s.) |
| T4 (pmol/l)  Norm: 9.0-25.0 | n = 176  8.9 ± 3.0 | n = 86  8.5 ± 2.4 | | n = 90  9.3 ± 3.4 | 0.149 (n.s.) |
