# Supplementary material for: Soy germ extract alleviates menopausal hot flushes: placebo-controlled double-blind trial
Source: Eur J Clin Nutr. 2018 May 30;72(7):961–70. doi: 10.1038/s41430-018-0173-3 (PMC6035149; doi:10.1038/s41430-018-0173-3)
Supplement: Supplementary file 2 — Supplementary Table 2 [file 41430_2018_173_MOESM2_ESM.docx]

Supplementary Table 2: Laboratory parameters with significant changes during the study. Values are presented for women completing the 24 weeks of treatment (n = 174: soy: n = 86; placebo: n = 90), excluding women with missing data.

|  | **Verum** | **Placebo** | **p** |
| --- | --- | --- | --- |
| Testosterone (Norm: < 3.5 nmol/l) | | | |
| n | 86 | 87 |  |
| Baseline | 0.9 ± 0.5 | 1.0 ± 0.5 | 0.047 |
| Wk 12 | 1.5 ± 0.7 | 1.1 ± 0.6 | **< 0.001** |
| Wk 24 | 1.6 + 0.7 | 1.3 ± 0.5 | **0.004** |
| Sexual Hormone Binding Globulin (Norm: 30-95 nmol/l) | | | |
| n | 86 | 88 |  |
| Baseline | 43.5 ± 16.8 | 60.2 ± 36.8 | **0.009** |
| Wk 12 | 47.3 ± 20.3 | 54.2 ± 29.4 | 0.154 |
| Wk 24 | 48.3 + 18.0 | 56.4 ± 31.5 | 0.249 |
| Iron (Norm: 7-28 µg/l) | | | |
| n | 86 | 90 |  |
| Baseline | 9.7 ± 5.3 | 9.8 ± 5.2 | 0.861 |
| Wk 12 | 10.4 ± 5.1 | 9.6 ± 4.6 | 0.415 |
| Wk 24 | 11.9 ± 5.0 | 9.9 ± 4.7 | **0,007** |
| Transferrin (Norm: 200-360 mg/dl) | | | |
| n | 85 | 88 |  |
| Baseline | 251.0 ± 31.6 | 269.1 ± 42.5 | **0.002** |
| Wk 12 | 252.0 ± 36.8 | 264.1 ± 45.8 | 0.042 |
| Wk 24 | 254.2 ± 40.9 | 264.2 ± 38.4 | 0.068 |
| Total cholesterol, subgroup < 250 mg/dl (= Norm) | | | |
| n | 60 | 67 |  |
| Baseline | 207 ± 26 | 208 ± 28 | 0.286 |
| Wk 12 | 206 ± 29 | 224 ± 32 | **< 0.001** |
| Wk 24 | 212 ± 34 | 212 ± 36 | 0.130 |
| Total cholesterol, subgroup ≥ 250 mg/dl | | | |
| n | 26 | 23 |  |
| Baseline | 298 ± 34 | 283 ± 23 | 0.066 |
| Wk 12 | 221 ± 42 | 266 ± 48 | **0.001** |
| Wk 24 | 214 ± 36 | 246 ± 27 | **0.001** |
| LDL cholesterol (Norm: ≤ 160 mg/dl) | | | |
| n | 86 | 89 |  |
| Baseline | 116 ± 35 | 120 ± 34 | 0.360 |
| Wk 12 | 113 ± 32 | 126 ± 26 | **0.003** |
| Wk 24 | 113 ± 34 | 116 ± 30 | 0.134 |
| Leukocytes (Norm: 4-11/nl) | | | |
| n | 85 | 90 |  |
| Baseline | 6.9 ± 1.1 | 6.9 ± 1.8 | 0.430 |
| Wk 12 | 7.0 ± 1.4 | 6.6 ± 1.6 | **0.004** |
| Wk 24 | 6.8 ± 1.5 | 6.5 ± 1.4 | 0.184 |
| Erythrocytes (Norm: 3.9-5.4/pl) | | | |
| n | 85 | 90 |  |
| Baseline | 4.4 ± 0.3 | 4.4 ± 0.3 | 0.858 |
| Wk 12 | 4.5 ± 0.3 | 4.4 ± 0.3 | **0.006** |
| Wk 24 | 4.5 ± 0.4 | 4.3 ± 0.5 | **< 0.001** |
| Hemoglobin (Norm: 12-16 g/dl) | | | |
| n | 85 | 90 |  |
| Baseline | 13.6 ± 0.9 | 13.5 ± 0.9 | 0.511 |
| Wk 12 | 13.8 ± 0.7 | 13.5 ± 1.0 | 0.133 |
| Wk 24 | 13.9 ± 0.7 | 13.4 ± 0.9 | **< 0.001** |
